# Concentration of Inorganic Elements Content in Benthic Seaweeds of Fernando de Noronha Archipelago by Synchrotron Radiation Total Reflection X-Ray Fluorescence Analysis (SRTXRF)

**DOI:** 10.1155/2012/407274

**Published:** 2012-02-08

**Authors:** Leandro De Santis Ferreira, Rosana Peporine Lopes, Mabel Norma Costas Ulbrich, Thais Guaratini, Pio Colepicolo, Norberto Peporine Lopes, Ricardo Clapis Garla, Eurico Cabral Oliveira Filho, Adrian Martin Pohlit, Orghêda Luiza Araújo Domingues Zucchi

**Affiliations:** ^1^Departamento de Física e Química, Faculdade de Ciências Farmacêuticas de Ribeirão Preto, Universidade de São Paulo, 14040-903 Ribeirão Preto, SP, Brazil; ^2^Instituto de Geociências, Universidade de São Paulo, 05508-080 São Paulo, SP, Brazil; ^3^Lychnoflora Pesquisa e Desenvolvimento em Produtos Naturais LTDA, Incubadora SUPERA, Campus da USP, 14040-900 Ribeirão Preto, SP, Brazil; ^4^Instituto de Química, Universidade de São Paulo, 05513-970 São Paulo, SP, Brazil; ^5^Departamento de Botânica, Ecologia e Zoologia, Centro de Biociências, Universidade Federal do Rio Grande do Norte, 59072-970 Natal, RN, Brazil; ^6^Instituto de Biociências, Universidade de São Paulo, 05508-090 São Paulo, SP, Brazil; ^7^Coordenação de Pesquisa em Produtos Naturais, Instituto Nacional de Pesquisa da Amazônia, 69060-001 Manaus, AM, Brazil

## Abstract

SRTXRF was used to determine As, Ba, Br, Ca, Co, Cr, Cs, Cu, Dy, Fe, K, Mn, Mo, Ni, Pb, Rb, Sr, Ti, V, and Zn in eleven seaweed species commonly found in Fernando de Noronha: *Caulerpa verticillata* (J. Agardh) (Chlorophyta), *Asparagopsis taxiformis* (Delile), *Dictyurus occidentalis* (J. Agardh), *Galaxaura rugosa* (J. Ellis & Solander) J. V. Lamouroux, *G. obtusata* (J. Ellis & Solander) J. V. Lamouroux, *G. marginata* (J. Ellis & Solander) J. V. Lamouroux (Rhodophyta), *Dictyota cervicornis* (Kützing), *Dictyopteris justii* (J. V. Lamouroux), *Dictyopteris plagiogramma* (Montagne) Vickers, *Padina gymnospora* (Kützing) Sonder, and a *Sargassum* sp. (Phaeophyta). Data obtained were compared to those from the analysis of other parts of the world seaweeds using different analytical techniques and were found to be in general agreement in terms of major and minor elemental components. Results provide baseline information about the absorption and accumulation of these elements by macroalgae in the area.

## 1. Introduction

In the South Atlantic Ocean is located Fernando de Noronha archipelago around 540 km of the northeastern Brazilian coast. This archipelago is composed by one large island and 20 small adjacent islets that represent a mountain chain top developed along an east-west fracture zone of the ocean floor and was built up by volcanic and subvolcanic essentially alkaline and subsaturated rocks [1].

The marine flora of Fernando de Noronha was first studied by Dickie [2]. Most of the investigations carried out since then were taxonomic studies [3–5]. Also, the families Dictyotaceae and Sargassaceae of brown algae, the green algae *Caulerpa verticillata*, and the red algae *Galaxaura* spp. are among the most abundant macroalgae on the rocky and reef shores of the archipelago [5, 6]. Their predominance is probably related to the production of secondary metabolites that inhibit herbivore predation [7].

Seaweeds require various mineral ions for photosynthesis and growth. Also, it has long been established that marine and estuarine macroalgae accumulate metals to levels many times those found in the surrounding waters [7], and several algae have been used for monitoring concentrations of elements [8–12]. This study provides baseline information for further investigations of the absorption and accumulation of 20 elements by eleven macroalgae species commonly found in Fernando de Noronha archipelago. The concentrations of the elements in the seaweeds were determined using Synchrotron Radiation Total Reflection X-Ray Fluorescence Analysis (SRTXRF).

## 2. Experimental

### 2.1. Chemicals Reagents and Solutions

All the reagents were purchased from Merck (Darmstadt, HE, Germany) and Synth (Diadema, SP, Brazil). The multielementary solution was prepared using monoelementary solutions purchased from Acros Organics (Geel, ANT, Belgium and New Jersey, NJ, USA), and ultrapure (deionized) water was obtained using a deionizer from Microtec (Ribeirão Preto, SP, Brazil).

### 2.2. Sampling

**Figure 1:**
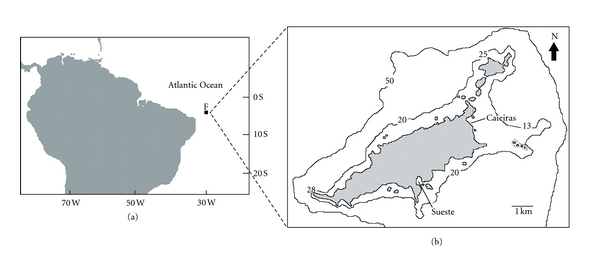
Geographical localization of study sites (Caieiras and Sueste) at the main island of Fernando de Noronha archipelago, off northeastern Brazil, where seaweeds were collected.

Eleven species of seaweeds commonly found in Fernando de Noronha archipelago were studied: *Caulerpa verticillata* (Chlorophyta), *Asparagopsis taxiformis*, *Dictyurus occidentalis*, *Galaxaura rugosa*, *G. obtusata*, *G. marginata* (Rhodophyta), *Dictyota cervicornis*, *Dictyopteris justii*, *Dictyopteris plagiogramma*, *Padina gymnospora,* and a *Sargassum* sp. (Phaeophyta) Samples were collected in February and March, 2006 at Caieiras Beach (3°50′18.8*″*S, 32°23′57.3*″*W) and Sueste Bay (3°52′1.2*″*S, 32°25′19.7*″*W) which are on the main island (Figure 1; Table 1). The IBAMA authorization to collect algae was registered with the number 050/2006. Seaweed specimens were collected randomly, that is, some individuals in a population were collect without a rule or defined sequence. Whole plants were uprooted and placed in labeled plastic bags. Seaweed samples were frozen and sent to the laboratory where they arrived 48 h after harvesting. The algae were identified by Prof. Dr. Eurico Cabral de Oliveira Filho. Residual sediment, epiphytes, and animals were removed, and the algae were washed with distilled water to remove seawater and air dried in a circulating air oven at 40°C for 48 h. After drying, around 5 g of each seaweed species was powdered by a triturating process in a grail after freezing the samples with liquid nitrogen. The powdered seaweeds were kept in a freezer until analysis was performed.

### 2.3. Sample and Calibration Solution Preparation

Samples of 250 mg of each algae species were placed in pyrex test tubes and digested according to a procedure described by Ward et al. [13]. Briefly, 6.0 mL of nitric acid (65%) and hydrogen peroxide (30%) were added to each test tube and homogenized. Test tubes were then placed on a digestion block overnight (*ca.* 12 hours) and heated at 130 ± 5°C until a translucid, particle-free, and fully digested solution was obtained. 5.00 mL of ultrapure water were transferred to the digested (sample) solution using a pyrex volumetric pipette, and the resulting solution was homogenized. The blank was a mixture of nitric acid, hydrogen peroxide, and deionized water and was done using the same procedure performed to the samples. Then 1.00 mL of this solution was removed using a pyrex volumetric pipette and to this aliquot was added 10 *μ*L of Ga (1.0 *μ*L mL^−1^) as an internal standard [14]. Calibration solutions of multielements that emit X-K and X-L rays were prepared, and Ga element was added as internal standard as above. 5.0 *μ*L of each sample were placed on a perspex support (polished quartz, 28 × 22 mm). The same procedure was done for 5.0 *μ*L of calibration solution. Drying for 1 h under, a 150 W infrared lamp (Phillips model 7, Amsterdam, NH, Netherlands) gave rise to a thin layer of approximately 5 mm diameter. Sample and calibration solutions were irradiated as described below. 

### 2.4. Instrumentation and Analysis Conditions

The equipment used was an X-ray fluorescence beamline constructed at the National Synchrotron Light Laboratory—LNLS (Campinas, São Paulo State, Brazil). For the total reflection of radiation, a series of mirrors are adjusted to allow that the synchrotron radiation hit the sample in small angle. The sample and calibration solutions were analyzed (three replicates of each) for 100 s each with a white synchrotron radiation beam using 0.5 mm of Al as absorber, 1.0 mm of Ta as collimator in the detector, a sample-to-detector distance of 1.1 mm, a height of 1 mm under total reflection conditions, and an angle of incidence of 1 mrad. The characteristic X-rays were detected with the aid of a Ge hyperpure semiconductor detector (resolution of 145 eV for energy of 5.9 keV).

### 2.5. Quantitative Analysis for X-Ray Fluorescence

Energy peaks detected in specters of the calibration samples were determined on the spectrometer, and the energy-emitting elements were identified from their X-ray characteristics (analytical lines). The liquid intensities for characteristic X-rays emitted were calculated with a mathematic adjustment in which the contribution of interfering lines on the analytical line (spectral interference) was considered including the escape peak and the addition peak. Mathematical adjustments were calculated with the software AXIL [15].

 In quantitative analysis, the fluorescent intensity of the characteristic line is related to the concentration by the expression *I*_*i*_ = *S*_*i*_ × *C*_*i*_ × *A*_*i*_, where *I*_*i*_ = fluorescent intensity of element *i* (cps), *S*_*i*_ = elemental sensitivity of element *i* (cps *μ*g^−1^ mL), *C*_*i*_ = concentration of element *i* (*μ*g mL^−1^), and *A* = absorption factor.

 Given the tiny thickness of the prepared samples, the absorption and/or intensification effects (matrix effect) of the analytical line are negligible. Thus, there is no need to consider the absorption factor [16], and the relation is *I*_*i*_ = *S*_*i*_ × *C*_*i*_.

 To correct like geometry and X-ray flow variation errors during excitation, Ga was used as internal standard. Ga is not present in the macroalgae samples. Referencing to internal standard yields the expression (*I*_*i*_/*I*_Ga_) = (*S*_*i*_/*S*_Ga_)×(*C*_*i*_/*C*_Ga_), where *I*_Ga_ = fluorescent intensity of Ga (cps), *S*_Ga_ = elemental sensitivity of Ga (cps *μ*g^−1^ mL), and *C*_Ga_ = concentration of Ga (*μ*g mL^−1^).

 If we define *S*_*i*_′ = *S*_*i*_/*S*_Ga_ and *R*_*i*_ = *C*_Ga_ × (*I*_*i*_/*I*_Ga_), where *S*_*i*_′  = relative sensitivity for element *i* (unidimensional) and *R*_*i*_ = product of relative intensity and *C*_Ga_ (*μ*g mL^−1^), then

(1) $$R_{i}=\;S_{i}^{\prime }\times C_{i}.$$

In the calibration solutions, *R*_*i*_ is directly proportional to *C*_*i*_; therefore, the angular coefficient of the calibration curve for the element *i* is its relative sensitivity. If *S*_*i*_^′  ^is known for the elements present in the calibration solutions, then the following function is obtained:

(2) $$\ln S_{i}^{\prime }=a+bZ_{i}+cZ_{i}^{2}+dZ_{i}^{3},$$

where *a*, *b*, *c*, and *d* are parameters that can be determinated by variance analyses, and *Z*_*i*_ is the atomic number for element *i*.

The relative sensitivity for any X-K or X-L ray emitting elements present in the samples can thus be calculated. The SANEST program was used to test significance of the parameters at 5% probability for inclusion in the model which was used to determine the above (2) [17]. The concentrations (*μ*g mL^−1^ or *μ*g g^−1^) for any inorganic element present in different samples were obtained from (1) after obtaining the experimental limits of detection (LD)(3)

(3) $${\text{LD}}_{i}=\frac{3\cdot \sqrt{{\text{BG}}_{i}/t}\cdot C_{\text{Ga}}}{I_{\text{Ga}}\cdot S_{i}^{\prime }},$$

where BG_*i*_ = background (cps), *t* = detection time (s), and other variables are defined above [18, 19].

 From the calculation of the experimental LD values, it was established that the values of LD are a polynomial function of the atomic number of the elements present, LD_*i*_ = *f*(*Z*_*i*_). Thus, using this formula, it is possible to calculate the LD for the elements which are not present in the sample.

## 3. Results and Discussion

**Figure 2:**
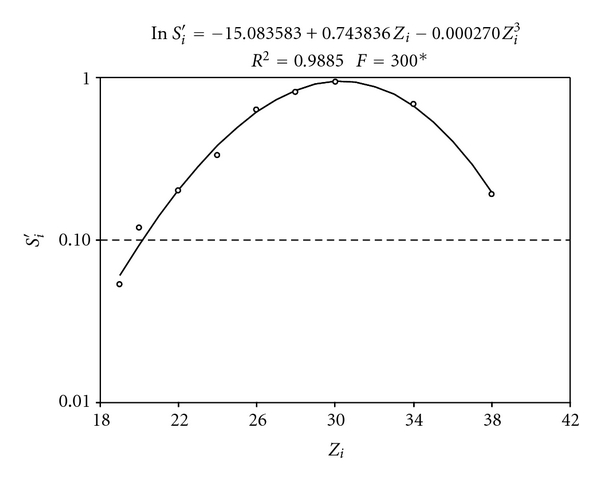
Experimental and calculated relative sensitivity for chemical elements emitting X-K rays for 19 ≤ *Z*_*i*_ ≤ 38.

**Figure 3:**
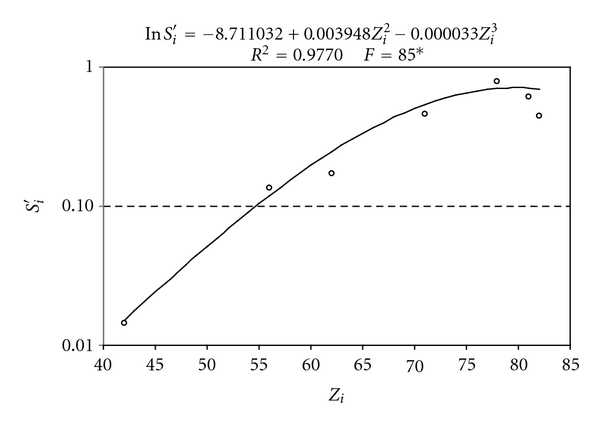
Experimental and calculated relative sensitivity for chemical elements emitting X-L rays for 42 ≤ *Z*_*i*_ ≤ 82.

Calibration curves (ln⁡*S*_*i*_′ = *f*(*Z*_*i*_)) with significant parameters at 5% level were obtained for all X-K (Table 2, Figure 2) and X-L ray (Table 3, Figure 3) emitting elements through the multielementary calibration solutions. For the SRTXRF technique, the maximum *S*_*i*_′ value obtained is for the internal standard which was the element Ga (*Z* = 31) in this experiment. The functions increase for *Z* < 31 until *Z* = 31 and decreasing for *Z* > 31. Theoretically, *S*_Ga_′ = 1.0; however, the experimental value of Ga was 0.93. It is important to correct for this difference in the calculation of the other elements. In this case, if the experimental data were used without experimental correction, an error of 6.6% can be observed for all the elements. Some authors attribute the need to perform these corrections to obtain the values close to true net intensities [20].

The minimum detection limits for X-K and X-L ray emitting elements are presented in Table 4. The lowest recorded detection limit for X-K ray emitting elements was LD = 0.01 ppm for Zn (*Z* = 30) and Ni (*Z* = 28) and the highest detection limit value was LD = 1.72 ppm for K (*Z* = 19). Lowest and highest detection limit values for X-L ray emitting elements were, respectively, 0.01 ppm for Cu (*Z* = 29) and 1.57 ppm for Mo (*Z* = 42). After determining the experimental detection limits, the concentration of each chemical element was estimated (Table 5). The results of the analysis of algae (Table 5) and the chemical composition of the alkaline rocks where the algae were collected (Table 6) were compared.

Essential elements Ca, Fe, K, Mn, and Zn were found in all algae samples as were relevant species CaO, Fe_2_O_3_, K_2_O, MnO, and Zn in the rocks upon which these algae grew. Interestingly, *G. marginata* had little Fe (75.27 ppm) while *D. plagiogramma* had over 150 times that amount (11936.65 ppm). *P. gymnospora* (3028.40 ppm) had the least values of Ca while *G. marginata* and *A. taxiformis* had the highest Ca levels detected (82606.32 and 88908.21 ppm, resp.). Relatively little K was found in *C. verticillata* (504.13 ppm) while the levels of K in *D. occidentalis* were almost 100 times this amount (49523.34 ppm). Also, the *P. gymnospora* had large amounts of Zn (274.44 ppm) whereas five algae species had levels of Zn in the range of 2–7 ppm Also, Sr was found in all rocks analyzed (*ca*. 950–1750 ppm) and was absorbed by all algae species and in relatively large abundance (*ca*. 500–6000 ppm). In contrast, Ba was generally abundant in rock samples (*ca*. 20–1350 ppm) from where the algae were collected, but Ba was only detected in *D. justii*. While Br was detected in several species, it was most concentrated in *A. taxiformis* (257.10 ppm).

The results obtained by SRTXRF analysis of algae are comparable to those obtained for algae from other parts of the world using other analytical methods. For example, Hou and Yan [22] studied elements present in 35 species of marine algae from the coast of China by neutron activation analysis in a miniature neutron source reactor (MNSR). They observed that, in brown, red, and green algae, the levels of individual elements were (averages, resp., in parentheses): As (159/<0.36/12.2 ppm), Ba (76.2/109.6/174 ppm), Br (3426/6157/596 ppm), Ca (22.7/29.7/11.2 ppt), Co (0.93/1.15/1.04 ppm), Cr (4.02/4.84/6.33 ppm), Cs (1.11/1.02/0.95 ppm), Fe (1892/2511/3716 ppm), K (67.5/48.4/29.0 ppt), Mn (857/89.4/90.6 ppm), Rb (29.4/21.5/25.8 ppm), Sr (892/313/161 ppm), and Zn (21.7/28.3/23.3 ppm). Besides this study, in another work about 26 marine benthic algae species found in Karachi Coast, Pakistan, the levels (averages for the 26 species are in parentheses) of Ca (26.75 ppt), Co (5.88 ppm), Cr (5.13 ppm), Cu (11.87 ppm), Fe (2.41 ppt), K (69.5 ppt), Pb (13.43 ppm), and Zn (53.28 ppm) were established using flame atomic absorption spectrometry (AAS) [23]. As in the algae in these previous studies, the algae of Fernando de Noronha were found to contain large amounts of Ca, K, and Fe (in parts per thousand, ppt) and small or trace amounts of As, Ba, Co, Cr, Cs, Cu, Pb, Rb, and Zn. Since data on the composition of the surfaces where algae in these previous studies grew was not reported, it is not possible to ascertain the contributions of these surface substrates to the composition of the algae. The similar compositional trends in principle and trace elements present in algae from these previous studies and those of Fernando de Noronha lends support to the usefulness of the SRTXRF technique in the analytical arsenal.

Low numbers of algae species contained Rb (4 algae species, 12–48 ppm), Ti (4 species, 109–537 ppm), and V (5 species, 14–44 ppm) which may be related to geological characteristics of the alkaline rocks present in the study sites. Low concentrations of As (5 species, range 11–118 ppm), Co (3 species, 1.9–5.4 ppm), Cr (6 species, range 5.3–54 ppm), Cu (3 species, range 1.1–1.6 ppm), Ni (4 species, range 1.9–15 ppm), and Pb (4 species, range 2.0–27 ppm) were observed in most of the algae species, except for *Sargassum* sp. which had a higher concentration of As (118 ppm) compared to the other species. These elements may have been absorbed from the seawater through natural weathering and lixiviation of rocks and soil. 

An interesting finding was the detection of Dy which is a rare earth metal present in rocks of both collection sites in *A. taxiformis, D. occidentalis, G. rugosa, G. obtusa, G. marginata, *and* D. plagiogramma*. Thus, these algae absorb and store this rare chemical element which is present in rocks in relatively low abundance.

## 4. Conclusion

 The SRTXRF technique proved to be adequate for the determination of 20 chemical elements present in eleven species of common macroalgae of Fernando de Noronha archipelago providing baseline information for the accumulation of metals in two sites. The results indicate a relationship among the metals present in the seaweeds and the rocks present in this area. Besides, the concentrations of common macro- and microelements obtained are comparable to those obtained by other authors using different analytical methods. However, multielement capability in a single analysis, high-sensitivity and precision, short analysis time, and easy sample preparation are some advantages of SRTXRF when compared to other elemental determination techniques such as AAS or ICP-MS.

## Figures and Tables

**Table 1:** Classification and sampling for eleven macroalgae studied in Fernando de Noronha archipelago, of northeastern Brazil.

Divisions and species	Site
Chlorophyta	
*Caulerpa verticillata* (J. Agardh, 1847)	S

Rhodophyta	
*Asparagopsis taxiformis *(Delile) Trevisan de Saint-Léon, 1845	S
*Dictyurus occidentalis* (J. Agardh, 1847)	C
*Galaxaura rugosa *(J. Ellis & Solander) J. V. Lamouroux, 1816	C
*Galaxaura obtusata* (J. Ellis & Solander) J. V. Lamouroux, 1816	C
*Galaxaura marginata *(J. Ellis & Solander) J. V. Lamouroux, 1816	C

Phaeophyta	
*Dictyota cervicornis* (Kützing, 1859)	S
*Dictyopteris justii* (J. V. Lamouroux, 1809)	S
*Dictyopteris plagiogramma *(Montagne) Vickers, 1905	C
*Padina gymnospora* (Kützing) Sonder, 1871	C
*Sargassum *sp. (C. Agardh, 1820)	S

S: Sueste; C: Caieiras.

**Table 2:** Experimental and calculated mean relative sensitivity, *S*_*i*_′, and mean standard deviation for chemical elements emitting X-K rays for 19 ≤ *Z*_*i*_ ≤ 38.

Element	**Z**_**i**_	**S**_**i**_^**'**^ (experimental)	**s**(**m**)^**a**^	**S**_**i**_^**'**^(calculated)
K	19	0.053007	0.004082	0.060650
Ca	20	0.118720	0.006506	0.093774
Ti	22	0.200669	0.015206	0.203081
Cr	24	0.329118	0.017632	0.381365
Fe	26	0.632468	0.027613	0.613014
Ni	28	0.810042	0.030792	0.832589
Zn	30	0.930767	0.025492	0.943175
Se	34	0.665939	0.051780	0.666817
Sr	38	0.190190	0.019045	0.195293

^*a*^ *s*(*m*) = standard deviation =$\;S_{i\;}^{\prime }(\text{exprimental})/\sqrt{n}$, *n* = 9.

**Table 3:** Experimental and calculated mean relative sensitivity,  *S*_*i*_′, and mean standard deviation for chemical elements emitting X-L rays for 42 ≤ *Z*_*i*_ ≤ 82.

Element	**Z**_**i**_	**S**_**i**_^**'**^ (experimental)	**s**(**m**)^**a**^	**S**_**i**_^**'**^(calculated)
Mo	42	0.014521	0.001182	0.015121
Ba	56	0.135400	0.008366	0.119381
Sm	62	0.170940	0.007042	0.246660
Lu	71	0.457106	0.014518	0.537822
Pt	78	0.789765	0.060052	0.743550
Tl	81	0.612795	0.047333	0.707681
Pb	82	0.445363	0.012872	0.697799

^*a*^ *s*(*m*) = standard deviation =$\;S_{i\;}^{\prime }(\text{exprimental})/\sqrt{n}$, *n* = 9.

**Table 4:** Minimum detection limits values (ppm) for X-K and X-L ray emitting elements.

Species	As	Ba	Br	Ca	Co	Cr	Cs	Cu	Dy	Fe	K	Mn	Mo	Ni	Pb	Rb	Sr	Ti	V	Zn
Chlorophyta																				
* C. verticillata*	0.02	—	0.03	0.29	—	0.03	—	—	—	0.02	0.36	0.02	—	—	—	—	0.09	0.05	0.04	0.01
Rhodophyta																				
* A. taxiformis*	**—**	—	0.08	0.90	—	0.09	—	—	0.12	0.07	1.12	0.08	—	—	0.06	—	0.21	0.18	0.13	0.03
* D. occidentalis*	0.02	—	—	0.22	—	0.03	0.12	0.01	0.03	0.02	1.72	0.03	0.43	—	—	0.05	0.08	—	—	0.01
* G. rugosa*	—	—	0.02	0.25	—	0.02	0.14	—	0.03	0.02	0.37	0.02	0.49	—	0.02	—	0.07	—	—	0.01
* G. obtusata*	—	—	—	0.40	—	—	0.22	—	0.05	0.03	0.60	0.04	0.86	—	—	—	0.11	—	—	0.01
* G. marginata*	—	—	—	0.87	—	—	0.46	—	0.12	0.07	1.35	0.08	1.57	—	—	—	0.23	—	—	0.02
Phaeophyta																				
* D. cervicornis*	—	—	0.07	0.73	—	0.08	—	—	—	0.06	1.26	0.07	—	—	0.06	—	0.21	0.18	0.11	0.03
* D. justii*	0.02	0.16	0.03	0.30	0.02	—	—	—	—	0.02	0.48	0.03	—	0.01	—	—	0.10	—	0.04	0.01
* D. plagiogramma*	—	—	—	0.83	—	0.08	0.30	—	0.13	0.08	1.16	0.08	—	0.05	0.07	0.16	0.25	0.11	—	0.05
* P. gymnospora*	0.01	—	0.02	0.09	0.01	—	0.06	0.01	—	0.01	0.21	0.01	—	0.01	—	0.03	0.04	—	—	0.01
* Sargassum *sp.	0.03	—	0.03	0.25	0.02	—	—	0.01	—	0.02	0.50	0.03	—	0.01	—	0.06	0.09	—	0.04	0.01

—**:** not calculated.

**Table 5:** Mean values of elemental composition (ppm) of eleven species of seaweeds collected in Fernando de Noronha Archipelago, off northeastern Brazil and their respective CV, coefficient of variation (%).

Species	As	Ba	Br	Ca	Co	Cr	Cs	Cu	Dy	Fe	K	Mn	Mo	Ni	Pb	Rb	Sr	Ti	V	Zn
Chlorophyta																				
*C. verticillata*	10.82 (11.26)	—	8.60 (13.63)	20639.78 (0.93)	—	11.91 (12.51)	—	—	—	2559.68 (0.47)	504.13 (5.08)	21.43 (6.40)	—	—	—	—	511.19 (1.80)	108.86 (3.66)	14.06 (8.37)	11.44 (3.83)
Rhodophyta																				
*A. taxiformis*	—	—	257.10 (2.64)	88908.21 (1.95)	—	25.87 (10.30)	—	—	108.47 (10.46)	4108.09 (1.77)	1988.31 (3.22)	77.14 (9.75)	—	—	12.18 (9.48)	—	2121.10 (1.79)	224.55 (4.35)	39.63 (5.53)	17.35 (1.61)
*D. occidentalis*	12.03 (8.43)	—	—	15366.59 (0.30)	—	6.26 (7.96)	62.98 (17.10)	1.55 (11.38)	6.12 (15.43)	915.92 (1.15)	49523.34 (1.29)	10.33 (4.35)	465.94 (14.12)	—	—	13.78 (10.85)	718.43 (2.12)	—	—	3.21 (18.63)
*G. rugosa*	—	—	1.65 (17.96)	22949.59 (1.63)	—	5.28 (2.41)	192.80 (5.05)	—	21.66 (15.51)	513.81 (2.55)	1092.76 (3.99)	15.64 (1.90)	832.84 (12.86)	—	2.00 (9.25)	—	1735.42 (3.63)	—	—	2.36 (12.17)
*G. obtusata*	—	—	—	41500.55 (6.75)	—	—	358.28 (7.76)	—	39.57 (14.48)	245.12 (5.92)	2611.29 (6.96)	23.58 (23.38)	671.37 (9.99)	—	—	—	3002.25 (9.42)	—	—	3.80 (16.81)
*G. marginata*	—	—	—	82606.32 (0.88)	—	—	736.05 (3.21)	—	54.34 (9.56)	75.27 (7.73)	15655.44 (0.62)	39.79 (5.66)	836.71 (7.84)	—	—	—	5957.57 (1.53)	—	—	5.70 (18.02)
Phaeophyta																				
*D. cervicornis*	—	—	20.21 (8.44)	44313.30 (1.04)	—	19.53 (5.43)	—	—	—	3621.88 (0.88)	40527.54 (1.21)	60.85 (4.33)	—	—	27.22 (1.73)	—	2272.40 (0.61)	181.51 (5.68)	44.39 (10.78)	25.54 (2.92)
*D. justii*	17.35 (2.37)	21.39 (14.88)	3.56 (12.04)	18394.89 (4.26)	3.53 (4.81)	—	—	—	—	1410.02 (4.05)	7370.06 (4.41)	30.33 (3.94)	—	5.35 (16.12)	—	—	3046.38 (4.15)	—	33.87 (3.00)	22.50 (1,67)
*D. plagiogramma*	—	—	—	56397.66 (2.17)	—	53.64 (7.44)	289.32 (15.38)	—	46.31 (19.97)	11936.65 (2.62)	10593.50 (2.35)	78.34 (6.44)	—	15.15 (5.23)	17.56 (16.64)	47.58 (12.20)	1521.78 (0.91)	537.01 (7.26)	—	24.58 (3.78)
*P. gymnospora*	15.20 (2.28)	—	8.03 (10.63)	3028.40 (0.55)	1.93 (17.99)	—	148.77 (2.46)	1.10 (11.52)	—	251.77 (0.73)	9910.06 (0.51)	7.04 (6.41)	—	1.91 (13.19)	—	12.75 (17.70)	881.64 (1.21)	—	—	274.44 (0.33)
*Sargassum *sp.	117.92 (0.10)	—	16.63 (5.92)	14035.01 (0.70)	5.35 (7.23)	—	—	1.61 (9.72)	—	172.12 (1.91)	27196.26 (0.84)	21.53 (5.85)	—	2.68 (8.54)	—	36.43 (9.32)	1453.50 (0.77)	—	14.13 (6.20)	6.15 (5.63)

Average	34.67	21.39	45.11	37103.66	3.60	20.41	298.03	1.42	46.08	2346.40	15179.33	35.09	701.71	6.27	14.74	27.63	2111.06	262.98	29.22	36.10

—: not detected.

**Table 6:** Representative chemical composition of alkaline rocks from Caieiras (1 to 5) and Sueste (6 to 8).

Caieiras					Sueste				
	1	2	3	4	5	6	7	8	Average
K_2_O^a^	1.45	4.85	4.86	2.92	1.83	2.21	5.16	3.67	3.37
CaO^a^	11.2	5.80	4.10	6.81	12.5	10.5	0.58	5.73	7.15
TiO_2_^^a^^	3.19	2.36	1.18	2.40	3.86	3.37	0.17	2.26	2.35
MnO^a^	0.18	0.18	0.15	0.21	0.16	0.21	0.19	0.12	0.18
Fe_2_O_3total_^a^	12.9	5.99	4.57	7.71	12.5	13.2	2.23	4.79	7.99
V^b^	275.1	164.0	71.0	171.8	239.9	257.0	13.0	187.4	172.40
Cr^b^	505.1	229.0	—	37.3	303.5	303.0	—	—	275.58
Co^b^	55.0	19.1	8.00	15.8	48.2	44.6	—	8.77	28.50
Ni^b^	358.9	23.6	17.9	62.4	295.5	309.6	—	19.6	155.36
Cu^b^	53.3	7.90	4.20	—	51.3	53.1	—	—	33.96
Zn^b^	57.5	97.0	95.3	94.3	122.5	114.1	181.0	80.0	105.21
Rb^b^	53.0	142.0	142.5	80.5	47.1	49.1	322.0	96.5	116.59
Sr^b^	960.9	820.8	1329	1620	1744	1023	—	1520	1288.24
Mo^b^	2.14	—	7.00	6.07	2.57	2.00	—	3.82	3.93
Cs^b^	1.20	1.90	2.40	3.67	0.55	1.00	—	4.33	2.15
Ba^b^	510.2	946.0	1191	1070	906.4	558.0	19.0	1330	816.33
Dy^b^	5.96	7.10	6.60	5.94	8.11	6.90	—	6.90	6.79
Pb^b^	—	13.3	14.7	14.5	5.21	7.20	32.0	11.9	14.12

—: not detected; ^a^wt %; ^b^ppm, —not analyzed. Using the procedure described in Janasi et al.[21], major, minor, and trace elements were determined by inductively coupled plasma-mass spectrometer (ICP-MS).
